# Serum 25-Hydroxyvitamin D Status and Longitudinal Changes in Weight and Waist Circumference: Influence of Genetic Predisposition to Adiposity

**DOI:** 10.1371/journal.pone.0153611

**Published:** 2016-04-14

**Authors:** Sofus C. Larsen, Lars Ängquist, Max Moldovan, Ville Huikari, Sylvain Sebert, Alana Cavadino, Tarunveer Singh Ahluwalia, Tea Skaaby, Allan Linneberg, Lise Lotte N. Husemoen, Ulla Toft, Oluf Pedersen, Torben Hansen, Karl-Heinz Herzig, Marjo-Riitta Jarvelin, Chris Power, Elina Hyppönen, Berit L. Heitmann, Thorkild I. A. Sørensen

**Affiliations:** 1 Research unit for Dietary Studies, the Parker Institute, Frederiksberg and Bispebjerg Hospitals, The Capital Region, Frederiksberg, Denmark; 2 Institute of Preventive Medicine, Bispebjerg and Frederiksberg Hospitals, the Capital Region, Copenhagen, Denmark; 3 Centre for Population Health Research, School of Health Sciences and Sansom Institute, University of South Australia, Adelaide, Australia; 4 Center for Life-Course Health Research, Faculty of Medicine, P.O.Box 5000, FI-90014 University of Oulu, Oulu, Finland; 5 Centre for Environmental and Preventive Medicine, Wolfson Institute of Preventive Medicine, Barts and the London School of Medicine and Dentistry, Queen Mary University of London, London, United Kingdom; 6 Population, Policy and Practice, UCL Institute of Child Health, University College London, London, United Kingdom; 7 The Novo Nordisk Foundation Center for Basic Metabolic Research, Section of Metabolic Genetics, Faculty of Health and Medical Sciences, University of Copenhagen, Copenhagen, Denmark; 8 Steno Diabetes Center, Gentofte, Denmark; 9 Research Centre for Prevention and Health, Capital Region of Denmark, Copenhagen, Denmark; 10 Department of Clinical Experimental Research, Rigshospitalet, Glostrup, Denmark; 11 Department of Clinical Medicine, Faculty of Health and Medical Sciences, University of Copenhagen, Copenhagen, Denmark; 12 Institute of Biomedicine, University of Oulu, Oulu, Finland; 13 Medical Research Center Oulu and Oulu University Hospital, Oulu, Finland; 14 Biocenter Oulu, P.O.Box 5000, Aapistie 5A, FI-90014 University of Oulu, Oulu, Finland; 15 Department of Gastroenterology and Metabolism, Poznan University of Medical Sciences, Poznan, Poland; 16 Department of Epidemiology and Biostatistics, MRC-PHE Centre for Environment and Health, School of Public Health, Imperial College London, London, United Kingdom; 17 Unit of Primary Care, Oulu University Hospital, Kajaanintie 50, P.O.Box 20, FI-90220 Oulu, 90029 OYS, Finland; 18 South Australian Health and Medical Research Institute, Adelaide, Australia; 19 The National Institute of Public Health, University of Southern Denmark, Copenhagen, Denmark; 20 The Boden Institute of Obesity, Nutrition, Exercise & Eating Disorders, University of Sydney, Sydney, Australia; 21 MRC Integrative Epidemiology Unit, University of Bristol, Bristol, United Kingdom; Weill Cornell Medical College in Qatar, QATAR

## Abstract

Studies of the relationship between serum 25-hydroxyvitamin D (25(OH)D) and changes in measures of adiposity have shown inconsistent results, and interaction with genetic predisposition to obesity has rarely been examined. We examined whether 25(OH)D was associated with subsequent annual changes in body weight (ΔBW) or waist circumference (ΔWC), and whether the associations were modified by genetic predisposition to a high BMI, WC or waist-hip ratio adjusted for BMI (WHR_BMI_). The study was based on 10,898 individuals from the Danish *Inter99*, the *1958 British Birth Cohort* and the *Northern Finland Birth Cohort 1966*. We combined 42 adiposity-associated Single Nucleotide Polymorphisms (SNPs) into four scores indicating genetic predisposition to BMI, WC and WHR_BMI_, or all three traits combined. Linear regression was used to examine the association between serum 25(OH)D and ΔBW or ΔWC, SNP-score × 25(OH)D interactions were examined, and results from the individual cohorts were meta-analyzed. In the meta-analyses, we found no evidence of an association between 25(OH)D and ΔBW (-9.4 gram/y per 10 nmol/L higher 25(OH)D [95% CI: -23.0, +4.3; P = 0.18]) or ΔWC (-0.06 mm/y per 10 nmol/L higher 25(OH)D [95% CI: -0.17, +0.06; P = 0.33]). Furthermore, we found no statistically significant interactions between the four SNP-scores and 25(OH)D in relation to ΔBW or ΔWC. Thus, in view of the narrow CIs, our results suggest that an association between 25(OH)D and changes in measures of adiposity is absent or marginal. Similarly, the study provided evidence that there is either no or very limited dependence on genetic predisposition to adiposity.

## Introduction

The high prevalence of vitamin D deficiency in developed countries [1;2] is considered a serious public health concern, and a growing body of evidence suggests that the biological mechanisms through which vitamin D exerts its actions goes far beyond regulation of bone mineralization and bone health [3–5]. Currently, vitamin D is suspected to play a role in the pathophysiology of several chronic diseases such as cardiovascular disease [6], hypertension [7], type 2 diabetes [8] and several forms of cancer [9]. Furthermore, cross-sectional studies have consistently shown an association between low serum 25-hydroxyvitamin D (25(OH)D) and obesity [10–13]. Moreover, vitamin D receptors are expressed in almost all tissues and organs [14]. Yet, the nature of the cross-sectional association between low vitamin D status and obesity remains unclear. Prospective studies examining the association between serum 25(OH)D and subsequent body weight (BW) change have shown inconsistent results [15–17]. However, results from a bi-directional Mendelian randomization study, based on 42,024 participants of European descent, suggested a causal relation for a higher BMI on reduced serum 25(OH)D status, while a general effect of serum 25(OH)D on BMI was likely to be absent or minimal [18]. Likewise, a recently published meta-analysis of 12 randomized trials, failed to show a statistically significant effect of oral vitamin D supplementation on BW change [19]. Nevertheless, a recently published longitudinal study of 796 children in the Brazilian Amazon observed a more pronounced association between *FTO* rs9939609 genotype and BW gain among children with a low vitamin D status [20]. Hence, an obesity preventive impact of vitamin D may be present among specific genetic subgroups.

During the last decade, genome-wide association studies (GWAS) have identified several common genetic variants associated with BMI, waist circumference (WC) or waist-hip ratio adjusted for BMI (WHR_BMI_) [21–33], and studies have suggested that different environmental factors may attenuate or enhance the impact that these genetic variants have on adiposity [34–36].

Given this background, the aim of our study was to examine whether serum 25(OH)D concentrations (nmol/L) were associated with subsequent annual changes in body weight (ΔBW; gram/y) or waist circumference (ΔWC; mm/y), and whether these associations were modified by genetic predisposition to a high BMI, WC or WHR_BMI_.

## Materials and Methods

### Study population

Our study was based on participants of white North-European descent from the Danish *Inter99* study, the *1958 British birth cohort* (*1958BC*), and the *Northern Finland Birth Cohort 1966* (*NFBC1966*).

*Inter99*: A multifactorial lifestyle intervention study (CT00289237, ClinicalTrials.gov), focusing on prevention of ischemic heart disease through healthy lifestyle. In 1999, an age- and sex- stratified random sample of 13,016 men and women born in 1939–40, 1944–45, 1949–50, 1954–55, 1959–60, 1964–65, 1969–70, living in 11 municipalities in the former Copenhagen County was drawn from the Civil Registration System and invited to a health examination. Of the 13,016 participants a total of 12,934 were eligible for invitation, and of these 6,784 participated [37;38]. In 2004, all participants from the baseline examination were re-invited for a follow-up study where the baseline examination programme was repeated [39]. Information on serum 25(OH)D concentrations was achieved from 6,497 participants. Of these, we had information on genetics, baseline and follow-up anthropometric measures as well as information on potential confounders on 4,437 individuals. For the present study, we excluded participants with non-Nordic descent (n = 158), and participants with prevalent cancer, cardiovascular disease or self-reported diabetes (n = 481) at baseline, ending up with 3,798 participants (3,295 in the analysis of ΔWC).

*1958BC*: In brief, participants were followed from birth to age 55 years. The original cohort consisted of all births (n = 17,638) born in one week of March 1958 in England, Scotland or Wales. At age 45 years, 11,971 participants were invited to attend a biomedical assessment and 9,377 took part [40]. For the present study we had information on serum 25(OH)D concentrations at age 45 years, BW at age 45 and 50 years (WC was only available at age 45y for the 1958BC), genetic information as well as information on potential baseline confounders on 4,276 individuals. Moreover, participants with self-reported diabetes at baseline (n = 209) were excluded from the analyses. Thus, we had a total of 4,067 participants, all of Caucasian descent.

*NFBC1966*: The cohort comprises a total of 12,058 live births to mothers living in the northern provinces of Finland, who were invited to participate if they were expected to deliver during 1966 [41]. Baseline information in the current study originates from the follow-up in 1998 (31 years of age), and information on follow-up of anthropometry was from 2013 (46 years of age). We had information on serum 25(OH)D concentrations at baseline, repeated measures of anthropometry, genetic information, as well as information on potential confounders on a total of 3,193 individuals. For the present study, we further excluded participants with prevalent cancer, cardiovascular disease or self-reported diabetes (n = 160). Thus, ending up with 3,033 individuals (2,916 in analyses of ΔWC).

All protocols in the three studies were in accordance with the Helsinki Declaration, were approved by the local Ethical Committees, and all participants provided written informed consent. The Inter99 study was approved by the Copenhagen County Ethical Committee (KA 98155) and registered in the Clinical Trials.gov (NCT00289237). The Northern Finland 1966 Birth Cohort was approved by the Ethical Committee of Oulu University Faculty of Medicine. For the 1958 British Birth Cohort, ethics approval for the study was obtained from the South-East Multi-Centre Research Ethics Committee (Ref: 01/1/44) and the Joint UCL/UCLH Committees on the Ethics of Human Research (Committee A: Ref: 08/H0714/40).

### 25-hydroxyvitamin D

In brief, *Inter99* blood measures were drawn at baseline after an overnight fast. Serum samples from the participants were stored at -20°C until 2010. Analysis of 25(OH)D was performed by high performance liquid chromatography [42]. In *1958BC*, fasting serum 25(OH)D concentrations were measured using an automated Immunodiagnostic Systems Limited OCTEIA assay with a Dade-Behring BEP2000 analyser and standardised according to the mean of Vitamin D Quality Assessment Scheme (DEQAS) [43]. In *NFBC1966*, total serum 25(OH)D was measured with high-performance liquid chromatography-tandem mass spectrometry [18]. In all cohorts, 25(OH)D was included in the analyses as a continuous variable (nmol/L) and as a categorical variable (insufficient: <50nmol/L and sufficient: ≥50nmol/L) [44]. Furthermore, season of blood sampling was included in analyses as a categorical variable (spring: Mar-May, summer: Jun-Aug, fall: Sep-Nov and winter: Dec-Feb).

#### SNP selection and genotyping

We had information on a total of 42 Single Nucleotide Polymorphism (SNPs) (genotyped, imputed or proxy-SNPs) shown to be consistently associated with BMI, WC or WHR_BMI_ at genome-wide significance levels in GWAS [21–33] (S1 Table).

The SNPs were successfully genotyped in *Inter99* using either the KASPar SNP genotyping method, or through human cardio-metabo bead chip array [45], and had a genotyping call rate >95%, an error rate ≤0.7% and showed no significant deviation from Hardy-Weinberg equilibrium (HWE) (P>0.05).

In *1958BC*, genome-wide data was obtained through two sub-studies. The first sub-study included 3,000 DNA samples randomly selected as part of the Welcome Trust Case Control Consortium (WTCCC2) and genotyped on the Affymetrix 6.0 platform. The second sub-study was a Type 1 diabetes case–control study (T1DGC) which used 2,500 DNA samples genotyped using the Illumina Infinium 550 K chip through the JDRF/WT Diabetes and Inflammation Laboratory (DIL) [46]. All SNPs genotyped had a call rate above >96% and showed no significant deviation from HWE (P > 0.05) except rs29941 (P = 0.001).

In *NFBC1966*, genomic DNA was extracted from whole blood using standard methods. Genotyping was performed using the Illumina Infinium 370cnvDuo array prepared for genotyping by the Broad Institute Biological Sample Repository (BSP) [46]. All genotyped SNPs had a call rate >99% and showed no significant deviation from HWE (P>0.05).

#### Genetic predisposition scores

For each individual, the 42 SNPs were coded 0, 1 or 2 according to the number of adiposity associated risk alleles. Four different SNP-scores were then calculated as indicators of genetic predisposition: scores based on summing risk alleles of all 42 SNPs (Composite-score), 26 BMI associated SNPs (BMI-score), 5 WC associated SNPs (WC-score) and 14 WHR_BMI_ associated SNPs (WHR-score), with higher scores indicating higher genetic predisposition to these specific traits, a method we have previously used to study gene × diet interaction in relation to changes in measures of adiposity [47–49].

### Anthropometric measures

Height and BW were measured at baseline and follow-up in all three cohorts. Weight was measured with the participants wearing indoor light clothing without shoes. WC was measured horizontally midway between the lower rib margin and the iliac crest in *Inter99* and *NFBC1966*. Follow-up measures on WC were not available in *1958BC*, and therefore analyses of ΔWC were not performed in this cohort. On the basis of these measures, we calculated changes in BW and WC in each cohort as the difference between baseline and follow-up. From this we calculated ΔBW (g/y) and ΔWC (mm/y) by dividing the derived differences (i.e. follow-up—baseline values) with the individual follow-up time in years.

### Covariates

Information on the participants’ baseline age and gender, and the women’s report on whether they had entered menopause were included as covariates. All participants reported information on baseline smoking status, and this information was included as a categorical variable: never smoked, former/occasional or current smoker. Likewise, all three cohorts had information about consumption of alcohol at baseline and this was included in the analyses as units of ethanol (1 unit = 1.5 cl or 12 g).

In *Inter99*, information on baseline physical activity was based on two questions on commuting physical activity and leisure time physical activity. From these two questions, overall physical activity was calculated by summing response on commuting physical activity (converted into minutes per week using a five-day working week) and a leisure time physical activity variable (converted into minutes per week) [39]. From this variable, overall physical activity was grouped into four categories <2 h/week, 2–3.9 h/week, 4–6.9 h/week and ≥7 h/week. In *1958BC* and *NFBC1966*, metabolic equivalent of task (MET) hours per week was calculated from reported frequencies and usual durations for several kind of activities [50;51], and included as a categorical variable (quartiles).

In *Inter99* and *NFBC1966*, education was assessed at baseline with questions about years of regular schooling, and for the present study the variable was classified as having a school education above the primary level or not. In *1958BC*, educational level was classified into five categories (none, some qualification, O-level, A-level or degree).

Finally, in *Inter99* we calculated participants´ total daily energy intake based on food frequency questionnaires. Total energy intake was included in supplemental analyses as a continuous variable (MJ/day).

### Statistical analyses

Linear regression was used to examine the association between serum 25(OH)D concentrations and subsequent ΔBW and ΔWC, with adjustments for height, baseline measure of outcome, age, sex, smoking status, education level, physical activity, season of 25(OH)D blood sample, alcohol consumption and menopausal status for women. All continuous variables were evaluated by model control (investigating linearity of effects on outcome(s), consistency with a normal distribution and variance homogeneity). Furthermore, we analyzed the association between SNP-scores and ΔBW and ΔWC among all participants, as well as 25(OH)D insufficient and sufficient participants separately.

To examine interaction between the four genetic predisposition scores and serum 25(OH)D concentration in relation to ΔBW or ΔWC, we correspondingly added the SNP-score variables as well as the interaction terms (SNP-score × 25(OH)D) to the dietary main effects-model. The calculated SNP-score × vitamin D interaction estimates can be conceptualized as their combined influence beyond the individual main associations. Furthermore, as Lourenco et al. found that *FTO* (rs9939609) interacts with 25(OH)D in relation to weight gain among children, we further investigated interaction between this particular variant and 25(OH)D in relation to ΔBW and ΔWC in our adult population [20].

After performing analyses in the individual cohorts, the results were combined through random effects meta-analyses, allowing for between-study heterogeneity. P-values ≤ 0.05 were regarded as statistically significant. Analyses were performed using the statistical software package Stata 12 (StataCorp LP, College Station, Texas, USA; www.stata.com).

### Supplementary analyses

For documentary and exploratory purposes, we performed the analysis of interactions between all the individual SNPs and serum 25(OH)D concentration in relation to ΔBW and ΔWC, with Bonferroni adjustment for multiple testing.

In *Inter99*, dietary information was available from food frequency questionnaires. Thus, to further limit the possibility of confounding by energy intake, we performed supplementary analyses adjusted for total energy intake. Furthermore, *Inter99* is a multifactorial lifestyle intervention study. Hence, we further adjusted the *Inter99* analyses for baseline intervention status. Moreover, to assess associations that were independent of ΔBW, the analysis with ΔWC as outcome was additionally performed with adjustment for concurrent ΔBW. This measure was also included instead of waist-hip ratio adjusted for BMI, as follow-up information on hip circumference was not available in all cohorts. The baseline correlation between waist-hip ratio adjusted for BMI and WC adjusted for BMI was 0.68 in Inter99.

## Results

For the current study, we had information on 10,898 individuals; 3,798 of whom were from *Inter99*, 4,067 from *1958BC*, and 3,033 from the *NFBC1966*. Information on 25(OH)D, anthropometry, SNP-scores and covariates are shown in Table 1. The highest baseline median 25(OH)D status was seen for the *1958BC* participants (57 nmol/L; 5^th^–95^th^ percentiles: 23–104 nmol/L) and the lowest among the *Inter99* participants (49 nmol/L; 5^th^–95^th^ percentiles: 16–102 nmol/L). Likewise, *1958BC* had the lowest proportion of participants with insufficient 25(OH)D status (39.1%), while *Inter99* had the highest (50.2%). Baseline median BW was lower in *NFBC1966* (69.9 kg; 5^th^–95^th^ percentiles: 52.0–96.4) than *Inter99* (76.0 kg; 5^th^–95^th^ percentiles: 55.0–104.1) and *1958BC* (77.2 kg; 5^th^–95^th^ percentiles: 55.4–107.1) which reflected the younger baseline age of *NFBC1966* participants. A similar pattern was seen for baseline WC. No substantial differences between the cohorts were seen for ΔBW or ΔWC.

**Table 1:** Information on 25-hydroxyvitamin D, anthropometrics, genetic predisposition scores and covariates in *Inter99*, *1958BC* and *NFBC1966*. Reported as median (5–95 percentiles) unless otherwise stated.

	*Inter99*	*1958BC*	*NFBC1966*
N	3,798	4,067	3,033
Follow-up time (years)	5.4 (5, 5.7)	5.0 (5, 5)	15.4 (14.6, 16.5)
**Basic variables**			
25(OH)D (nmol/L)	49 (16, 102)	57 (23, 104)	50 (29, 76)
25(OH)D (% insufficient)^1^	50.2	39.1	49.6
Gender, %women	51.1	46.4	56.5
Baseline age (years)	45.1 (34.7, 59.8)	45 (45, 45)	31.1 (30.5, 31.7)
Height (cm) ^2^	172.0 (158.0, 187.5)	170.1 (155.2, 185,0)	170.3 (157,0, 186,0)
**BW** ^3^			
Baseline (kg)	76.0 (55.0, 104.1)	77.2 (55.4, 107.1)	69.9 (52.0, 96.4)
Follow-up (kg)	76.7 (56.0, 105.1)	77.6 (54.4, 107.9)	76.5 (55.8, 107.7)
ΔBW (kg/y)	0.2 (-1.4, 1.8)	0.1 (-1.8, 2)	0.4 (-0.3, 1.4)
**WC** ^4^			
Baseline (cm)	85.0 (67.0, 107.0)	-	82.0 (66.5, 104.0)
Follow-up (cm)	88.0 (69.0, 110.0)	-	90.0 (72.0, 115.0)
ΔWC (cm/y)	0.5 (-1.3, 2.4)	-	0.5 (-0.3, 1.5)
**SNP-based variables** ^5^			
BMI-score	24 (19, 30)	24 (19, 30)	24 (19, 30)
WC-score	3 (1, 5)	3 (1, 5)	3 (1, 5)
WHR-score	14 (10, 18)	14 (10, 18)	15 (11, 19)
Composite-score	39 (32, 46)	39 (32, 46)	40 (34, 47)
**Adjustment variables**			
Smoking, % Never smokers	40.0	22.2	27.7
Education, % ≤ Primary school	26.2	21.2	56.7
Physical activity, % most sedentary group	11.0	23.0	22.8
Menopausal status, % postmenopausal	27.9	6.7	0.0
Alcohol (Units/day)	0.8 (0.0, 4.4)	0.7 (0.7, 2.6)	0.3 (0.0, 2.6)
**Season of 25(OH)D blood sample %**			
Mar-May	30.3	19.2	21.5
Jun-Aug	23.0	23.3	38.3
Sep-Nov	26.1	40.1	29.2
Dec-Feb	20.7	17.5	11.0

Abbreviations: 25(OH)D, 25-hydroxyvitamin D; BW, body weight; WC, waist circumference; ΔBW, annual body weight change; ΔWC, annual waist circumference change; HRT, hormone replacement therapy.

^1^ 25(OH)D status: Insufficient, 25(OH)D <50nmol/L.

^2^ Gender-specific medians (5–95 percentiles). For male/woman in Inter99 baseline height (179/166 cm [168/156, 191/177]). For male/woman in 1958BC baseline height (176/163 cm [166/153, 188/173]). For male/woman in 1958BC baseline height (178/165 cm [168/155, 189/175])

^3^ Gender-specific medians (5–95 percentiles). For males/woman in Inter99 baseline BW (84/68 kg [66/53, 108/96]), follow-up BW (85/69 kg [66/53, 108/96]), ΔBW (0.2/0.2 kg [-1.3/-1.5, 1.8/1.7]). For males/woman in 1958BC baseline BW (85/67 kg [65/52, 110/99]), follow-up BW (86/67 kg [66/52, 114/97]), ΔBW (0.1/-0.0 kg [-1.6/-2.1, 2.1/2.0]). For males/woman in NFBC1966 baseline BW (79/63 kg [63/51, 101/89]), follow-up BW (85/69 kg [67/54, 113/100]), ΔBW (0.4/0.4 kg [-0.3/-0.3, 1.3/1.4]).

^4^ Inter99: n = 3,295 on ΔWC. NFBC1966: n = 2,916 on ΔWC. Gender-specific medians (5–95 percentiles). For males/woman in Inter99 baseline WC (91/77 cm [77/64, 110/101]), Follow-up WC (94/80 cm [79/67, 112/104]), ΔWC (0.4/0.7 cm/y [-1.3/-1.3, 2.2/2.5]). For males in NFBC1966 baseline WC (88/76 cm [75/65, 106/101]), Follow-up WC (96/86 cm [81/70, 118/112]), ΔWC (0.5/0.5 cm/y [-0.2/-0.3, 1.4/1.6]).

^5^ Sum of BMI, WC or WHRBMI associated risk-alleles. In Inter99: n = 2,605 on BMI-score, n = 3,272 on WC-score, n = 3,058 on WHR-score, n = 2,162 on Composite-score. In 1958BC: n = 3,900 on BMI-score, n = 4,067 on WC-score, n = 4,066 on WHR-score, n = 3,899 on Composite-score. In NFBC1966: n = 3,005 on BMI-score, n = 3,030 on WC-score, n = 3,018 on WHR-score, n = 2,990 on Composite-score.

Information on the 42 included SNPs along with information on which adiposity traits they have been a priori associated with can be found in S1 Table.

### Vitamin D status and subsequent change in anthropometry

**Fig 1:**
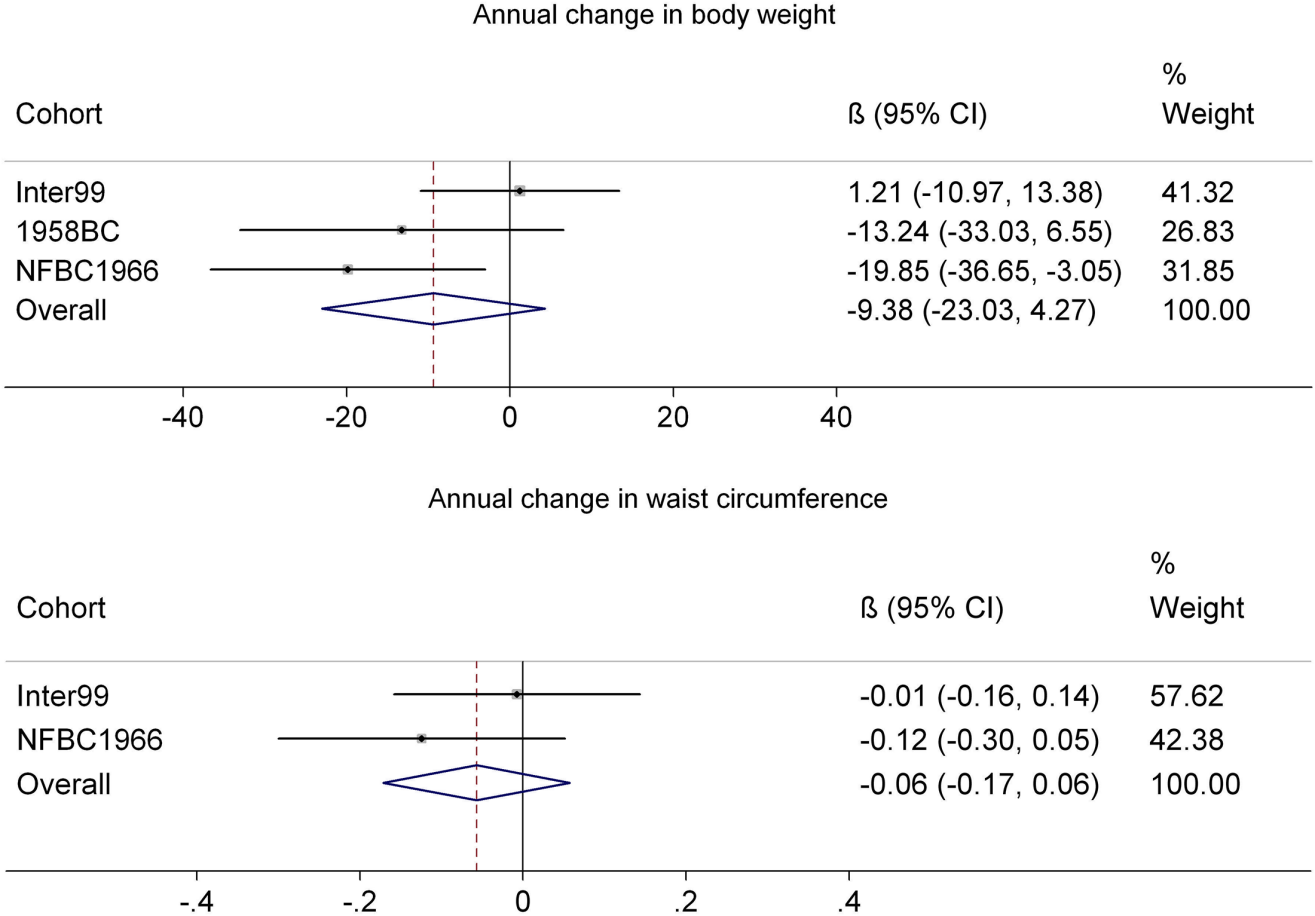
Annual change in body weight (g/y) and waist circumference (mm/y) per 10 nmol/L higher 25-hydroxyvitamin D status. Estimates were calculated in Inter99, 1958BC and NFBC1966 using linear regression and the results were subsequently meta-analyzed using a random effects approach. Adjusted for baseline outcome, height, gender, age, smoking status, alcohol consumption, physical activity, education, menopausal status for women and season of blood draw.

The meta-analysis of the association estimates between serum concentration of 25(OH)D and ΔBW and ΔWC are shown in Fig 1. The analysis of the NFBC1966 showed that 25(OH)D at baseline was associated with ΔBW (-19.85 gram/y; 95% CI: -36.65, -3.05; P = 0.02 per 10 nmol/L increase in 25(OH)D). However, this association was neither replicated in the other populations studied, nor in the meta-analysis of the three cohorts (-9.38 gram/y; 95% CI: -23.03, +4.27; P = 0.18 per 10 nmol/L higher 25(OH)D).

Similarly, the analyses with ΔWC as the outcome, did not reject the null hypothesis and no detectable associations were found in *Inter99*, *NFBC1966* or in the meta-analysis including both cohorts (-0.06 mm/y (95% CI: -0.17, +0.06; P = 0.33) per 10 nmol/L higher 25(OH)D).

### SNP-scores and changes in anthropometry stratified by vitamin D status

Table 2 shows the association between the four SNP-scores and ΔBW among all participants, and stratified on baseline 25(OH)D status (insufficient: <50 nmol/L and sufficient: ≥50 nmol/L). No evidence for an association was observed between the BMI-score, the WC-score or the Composite-score and ΔBW. Likewise, no significant difference was observed between 25(OH)D insufficient and sufficient participants. In the meta-analysis of all three cohorts each additional risk allele from the WHR-score was associated with a ΔBW of -9.5 gram/y (-16.1, -2.8; P = 0.004). The same pattern was found for both 25(OH)D sufficient and insufficient participants, with no significant differences between the groups (P for interaction = 0.873).

**Table 2:** Association between SNP-scores and annual weight change (gram/y) stratified by baseline vitamin D status.

	All		Baseline vitamin D status		
	N	β (95% CI)^1^	Insufficient^2^ β (95% CI)	Sufficient β (95% CI)	P-interaction
***Inter99***					
BMI-score	2,605	1.4 (-9.8, 12.5)	3.0 (-13.4, 19.4)	-1.3 (-16.4, 13.8)	0.671
WC-score	3,272	3.3 (-20.2, 26.9)	-15.5 (-50.7, 19.6)	25.3 (-6.1, 56.7)	0.061
WHR-score	3,058	-13.3 (-27.6, 1.0)	-9.8 (-31.5, 11.9)	-14.7 (-33.4, 4.0)	0.796
Composite-score	2,162	-6.2 (-15.9, 3.4)	-2.8 (-17.0, 11.3)	-8.9 (-22.2, 4.4)	0.657
***1958BC***					
BMI-score	3,900	7.5 (-6.2, 21.3)	4.6 (-19.6, 28.8)	10.6 (-5.9, 27.1)	0.859
WC-score	4,067	15.2 (-16.0, 46.4)	27.5 (-25.9, 80.8)	9.3 (-28.9, 47.4)	0.426
WHR-score	4,066	-13.8 (-31.8, 4.2)	-19.0 (-50.9, 13.0)	-11.4 (-33.0, 10.2)	0.657
Composite-score	3,899	-1.8 (-12.4, 8.7)	-3.8 (-22.3, 14.8)	0.2 (-12.5, 12.9)	0.848
***NFBC1966***					
BMI-score	3,005	3.5 (-2.5, 9.5)	4.3 (-4.5, 13.3)	3.1 (-5.1, 11.2)	0.819
WC-score	3,030	10.6 (-3.6, 24.8)	18.6 (-2.6, 39.8)	2.2 (-16.8, 21.2)	0.232
WHR-score	3,018	-7.2 (-15.5, 1.0)	-8.0 (-20.3, 4.4)	-6.9 (-17.9, 4.1)	0.880
Composite-score	2,990	-0.1 (-4.8, 4.6)	1.0 (-6.1, 7.9)	-1.1 (-7.4, 5.2)	0.692
**Meta-analyses** ^3^					
BMI-score	9,510	3.6 (-1.3, 8.6)	4.1 (-3.3, 11.5)	3.4 (-3.1, 10.0)	0.752
WC-score	10,369	9.6 (-1.8, 20.9)	11.3 (-5.9, 28.5)	8.5 (-6.5, 23.5)	0.741
WHR-score	10,142	-9.5 (-16.1, -2.8)	-9.5 (-19.7, 0.7)	-9.3 (-18.0, -0.7)	0.873
Composite-score	9,051	-1.4 (-5.3, 2.6)	-0.2 (-6.1, 5.7)	-2.1 (-7.3, 3.1)	0.657

Abbreviations: SNP-score (BMI, WC, WHR or Composite), sum of BMI, waist circumference or waist-hip ratio adjusted for BMI associated risk-alleles.

^1^ Adjusted for baseline body weight, height, gender, age, smoking status, alcohol consumption, physical activity, education, menopausal status for women and season of blood draw.

^2^ 25-hydroxyvitamin D <50nmol/L.

^3^ Estimates were calculated in Inter99, 1958BC and NFBC1966 using linear regression and the results were subsequently meta-analyzed using a random effects approach.

In analyses of all participants, we found no association between the four SNP-scores and ΔWC. Some indication for differences in the association between the WC-score and ΔWC was observed for 25(OH)D insufficient and sufficient participants in both *Inter99* and *NFBC1966*. However, the direction of the observed interactions were opposite in the two cohorts. In *Inter99*, we found an association between the WC-score and a higher ΔWC among 25(OH)D sufficient, while no association was observed among 25(OH)D insufficient participants. In contrast, results from *NFBC1966* showed that the WC-score was associated with a lower ΔWC among 25(OH)D insufficient participants, while no association was observed among 25(OH)D sufficient individuals. Thus, in the meta-analysis no difference was observed between 25(OH)D insufficient and sufficient participants (P for interaction = 0.399) (Table 3).

**Table 3:** Association between SNP-scores and annual change in waist circumference (mm/y) stratified by baseline vitamin D status.

	All		Baseline vitamin D status		
	N	β (95% CI) ^1^	Insufficient ^2^ β (95% CI)	Sufficient β (95% CI)	P-interaction
***Inter99***					
BMI-score	2,243	0.01 (-0.13, 0.16)	0.02 (-0.18, 0.23)	-0.05 (-0.24, 0.15)	0.603
WC-score	2,830	0.10 (-0.20, 0.39)	-0.30 (-0.73, 0.13)	0.45 (0.05, 0.86)	0.008
WHR-score	2,652	-0.05 (-0.23, 0.12)	-0.02 (-0.28, 0.23)	-0.11 (-0.35, 0.13)	0.706
Composite-score	1,863	-0.00 (-0.13, 0.12)	0.04 (-0.14, 0.21)	-0.07 (-0.24, 0.10)	0.472
***NFBC1966***					
BMI-score	2,889	0.05 (-0.01, 0.11)	0.08 (-0.01, 0.17)	0.02 (-0.06, 0.11)	0.465
WC-score	2,913	0.14 (-0.01, 0.29)	0.30 (0.08, 0.52)	-0.04 (-0.24, 0.16)	0.023
WHR-score	2,902	-0.06 (-0.15, 0.03)	0.06 (-0.19, 0.07)	-0.06 (-0.18, 0.05)	0.948
Composite-score	2,875	0.01 (-0.04, 0.06)	0.04 (-0.03, 0.11)	-0.01 (-0.08, 0.05)	0.279
**Meta-analyses** ^3^					
BMI-score	5,132	0.04 (-0.01, 0.10)	0.07 (-0.02, 0.15)	0.01 (-0.07, 0.09)	0.326
WC-score	5,743	0.13 (-0.00, 0.26)	0.18 (-0.02, 0.37)	0.06 (-0.12, 0.24)	0.399
WHR-score	5,554	-0.06 (-0.14, 0.02)	-0.05 (-0.17, 0.06)	-0.07 (-0.18, 0.03)	0.974
Composite-score	4,738	0.01 (-0.03, 0.06)	0.04 (-0.03, 0.11)	-0.02 (-0.08, 0.04)	0.358

Abbreviations: SNP-score (BMI, WC, WHR or Composite), sum of BMI, waist circumference or waist-hip ratio adjusted for BMI associated risk-alleles.

^1^ Adjusted for baseline waist circumference, height, gender, age, smoking status, alcohol consumption, physical activity, education, menopausal status for women and season of blood draw.

^2^ 25-hydroxyvitamin D <50nmol/L.

^3^ Estimates were calculated in Inter99 and NFBC1966 using linear regression and the results were subsequently meta-analyzed using a random effects approach.

### Interaction between genetic predisposition scores and serum 25-hydroxyvitamin D

**Fig 2:**
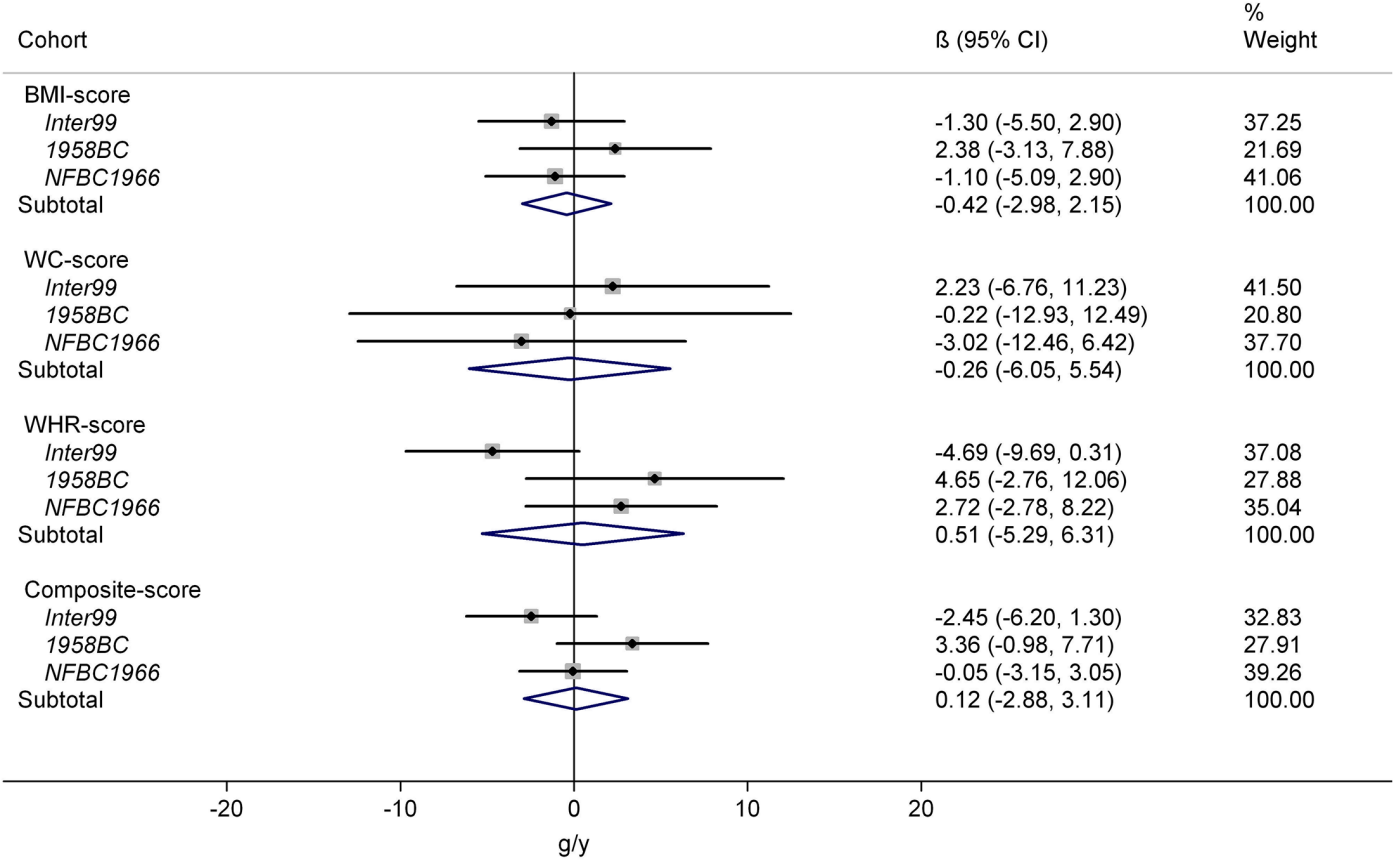
Interaction between genetic predisposition scores and 25-hydroxyvitamin D in relation to subsequent change in body weight. Abbreviations: BMI score, sum of body mass index associated risk-alleles; WC score, sum of waist circumference associated risk-alleles; WHR score, sum of waist-hip ratio adjusted for BMI associated risk-alleles; Composite score, sum of SNP associated to all three phenotypes. Results presented as annual weight change (g/y) effect-modification for each additional risk-allele per 10 nmol/L higher 25-hydroxyvitamin D. The study-specific SNP-score × 25-hydroxyvitamin D interactions were calculated using linear regression and corresponding meta-analysis results were derived using a random effects approach. The results were adjusted for baseline measure of body weight, height, gender, age, smoking status, alcohol consumption, physical activity, education, menopausal status for women and season of blood draw.

No significant interactions were observed between the four SNP-scores and 25(OH)D in relation to ΔBW in the individual cohorts or in the meta-analyses (Fig 2).

**Fig 3:**
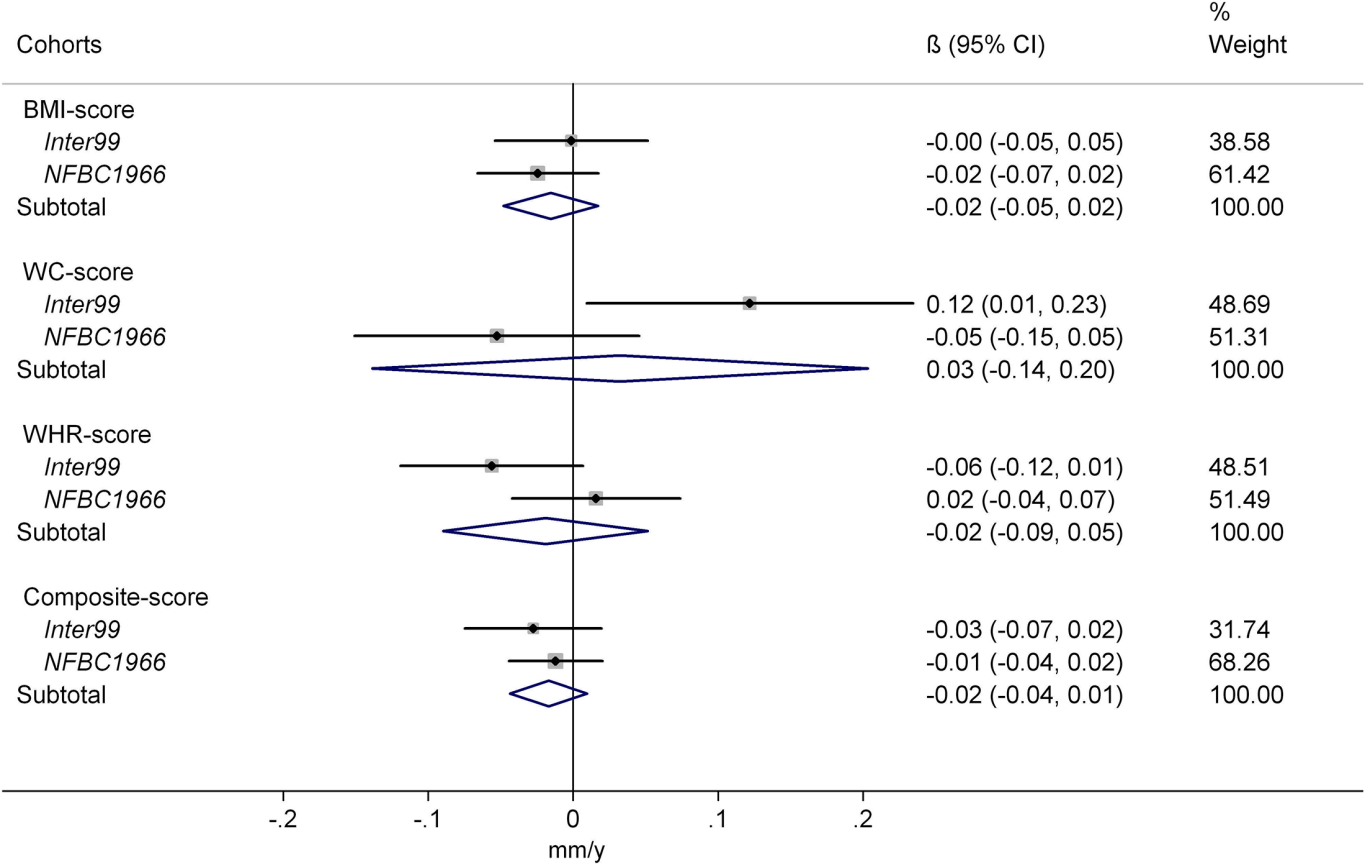
Interaction between genetic predisposition scores and 25-hydroxyvitamin D in relation to subsequent change in waist circumference. Abbreviations: BMI score, sum of body mass index associated risk-alleles; WC score, sum of waist circumference associated risk-alleles; WHR score, sum of waist-hip ratio adjusted for BMI associated risk-alleles; Composite score, sum of SNP associated to all three phenotypes. Results presented as annual change in waist circumference (mm/y) effect-modification for each additional risk-allele per 10 nmol/L higher 25-hydroxyvitamin D. The study-specific SNP-score × 25-hydroxyvitamin D interactions were calculated using linear regression and corresponding meta-analysis results were derived using a random effects approach. The results were adjusted for baseline measure of waist circumference, height, gender, age, smoking status, alcohol consumption, physical activity, education, menopausal status for women and season of blood draw.

In *Inter99*, the WC-score appeared to modify the association between 25(OH)D and ΔWC, while in the *NFBC1966* cohort no interaction was observed, and the meta-analyzed product term was not statistically significant (Fig 3).

### Interaction between FTO (rs9939609) and serum 25-hydroxyvitamin D

**Fig 4:**
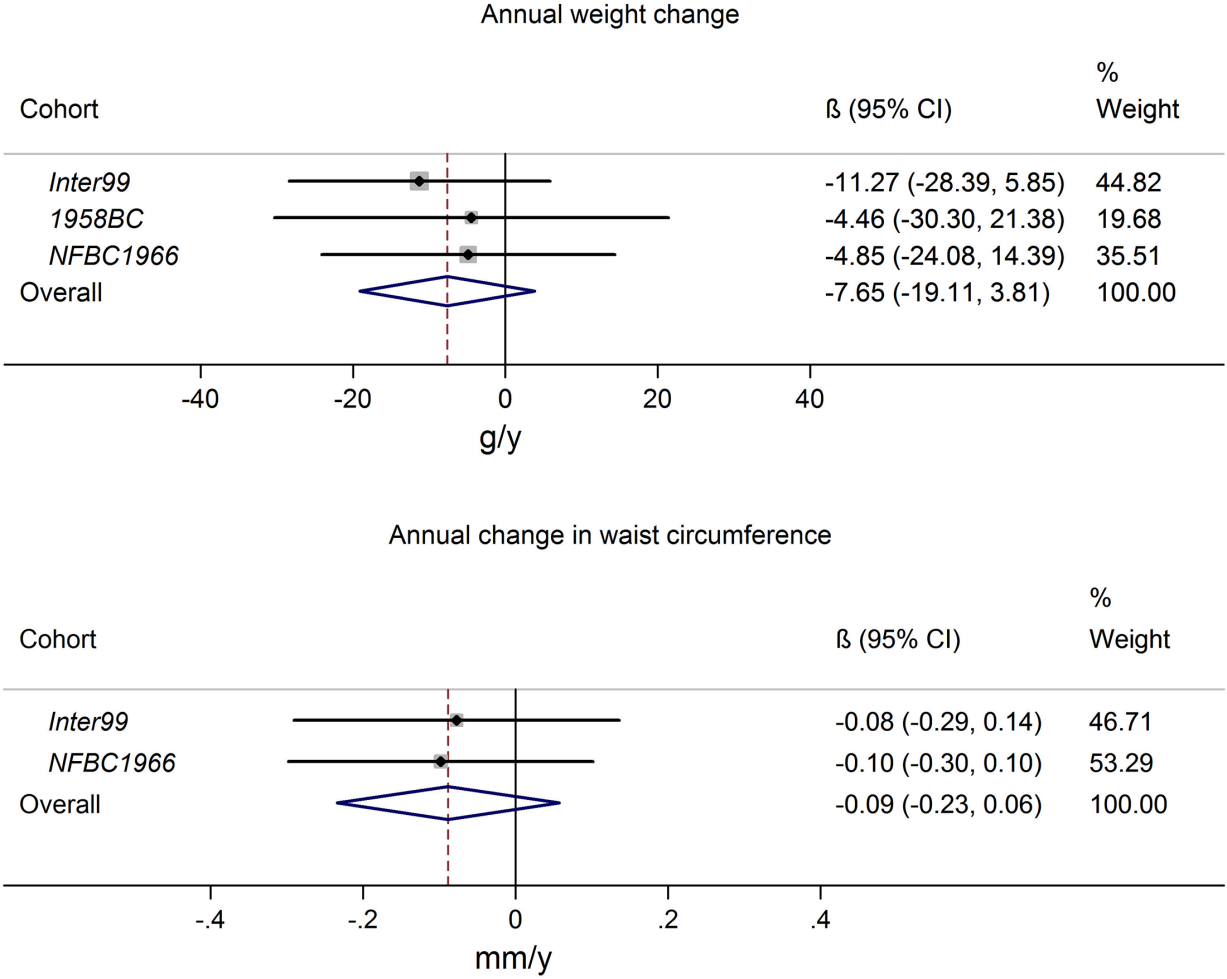
Interaction between FTO (rs9939609) and 25-hydroxyvitamin D in relation to subsequent change in body weight and waist circumference Results presented as annual change in body weight (g/y) and waist circumference (mm/y) effect-modification for each additional A-allele per 10 nmol/L higher 25-hydroxyvitamin D. The study-specific FTO × 25-hydroxyvitamin D interactions were calculated using linear regression and corresponding meta-analysis results were derived using a random effects approach. The results were adjusted for baseline measure of exposure, height, gender, age, smoking status, alcohol consumption, physical activity, education, menopausal status for women and season of blood draw.

The direction of the interaction estimates was fairly consistent for the three cohorts, indicating that the *FTO* risk allele was associated with a slightly lower gain in BW and WC among participants with higher serum 25(OH)D, consistent with the results among children published by Lourenco et al. [20]. However, the results from meta-analyses were not statistically significant (Fig 4).

### Supplementary analyses

We also examined interaction between the 42 individual SNPs and 25(OH)D in relation to ΔBW (S2 Table) and ΔWC (S3 Table). However, there was no evidence for interaction between any of the 42 SNPs and 25(OH)D after Bonferroni adjustment for multiple testing.

Further adjustment for total energy intake and baseline intervention status in the *Inter99* cohort had only negligible influence on the observed main effect of 25(OH)D on change in anthropometry (S4 Table) or on the SNP-score × 25(OH)D interaction estimates (S5 Table). Likewise, further adjusting the analyses of ΔWC for concurrent ΔBW in *Inter99* and *NFBC1966* had no substantial influence on the observed associations (S1 and S2 Figs).

## Discussion

In analyses of up to 10,898 individuals from three prospective European cohorts with longitudinal follow-up, we found no significant associations between 25(OH)D and ΔBW or ΔWC, and the corresponding confidence intervals were quite narrow. Likewise, our analyses did not suggest that there are associations between 25(OH)D and ΔBW or ΔWC that are modified by scores of genetic variants related to BMI, WC or WHR_BMI_.

Although no evidence of association was observed in these meta-analyzed results, some differences were seen in the individual cohorts. For example, there was an association between higher 25(OH)D and subsequent lower BW gain in the Finnish *NFBC1966*, but no association was observed in the Danish *Inter99* and the British *1958BC*. Another heterogeneity was observed in the results for interaction between the WC-score and 25(OH)D in relation to ΔWC. In fact, while the analysis from the *Inter99* indicated that 25(OH)D was associated with a greater increase in WC with higher genetic predisposition score, the opposite trend was observed in the *NFBC1966*.

While some prospective cohort studies have indicated a link between higher serum 25(OH)D and less BW gain or incident obesity [15;16], other studies have suggested that such a relationship is not present [17;18]. Furthermore, the previously mentioned meta-analysis of 12 randomized controlled trials found no evidence for a protective effect of vitamin D supplementation on change in BMI [19]. Thus, our study builds on evidence that any causal relationship between 25(OH)D and BW gain is either not present or small. Previous studies investigating the association between 25(OH)D and subsequent change in adiposity measures have generally not taken into account possible interaction with genetic predisposition to obesity. However, as mentioned previously, Lourenco et al. (2014) found an interaction between *FTO* (rs9939609) and 25(OH)D in relation to subsequent BW gain in childhood [20]. We were unable to replicate an interaction between *FTO* and 25(OH)D in relation to ΔBW in our adult population (P = 0.19) suggesting that this might be an age-specific interaction. However, in our study the direction of the interaction estimates, although not statistically significant, were consistent across all cohorts. Therefore, vitamin D status may be associated with a lower BW or WC gain per additional *FTO* risk allele, which corresponds to the direction of the interaction observed by Lourenco et al. (20)

The main strengths of our study are the use of data from three large cohorts with information on 25(OH)D, with a wide-range of serum concentrations and an equal distribution of 25(OH)D sufficient and insufficient participants, repeated measures of anthropometry as well as information on several potential confounders. Likewise, we had information on 42 SNPs found to be consistently associated with different adiposity measures in GWAS, allowing us to calculate genetic predisposition scores. Furthermore, we conducted prospective analyses of changes in anthropometry limiting the influence of reverse causality.

However, our study has some limitations. We relied one a single baseline measure of 25(OH)D, but repeated exposure measurements during the follow-up period would have strengthened our study. Estimated 25(OH)D concentrations from commercially available assays are related to some uncertainty. Moreover, we used different methods to measure 25(OH)D in the three cohorts. Although this may lead to slightly different concentration estimates in the three cohorts, it is unlikely to have a substantial influence on the overall results, as the most important factor in this context is the methods ability to rank individuals correctly. In the present study, we used changes in BW and WC as outcomes which are not the most sensitive measures of adiposity, especially considering the small annual changes observed in the present study, and this may partly explain the limited findings.

Furthermore, as mentioned, some dissimilarity in the results was observed between the cohorts. While this is likely due to chance findings, we are unable to rule out influence of design differences between the three cohorts. In this regard, both *Inter99* and *1958BC* had an average follow-up time of roughly 5 years, while the follow-up time in *NFBC1966* was approximately 15 years. Another important consideration is the different baseline ages in the three cohorts. While the baseline median age for *Inter99* and *1958BC* was approximately 45 years, *NFBC1966* participants were approximately 31 years old, and we cannot rule out the possibility that the influence of vitamin D on the adiposity measures varies with age. Differences due to geographic location of the included cohorts or country-specific gene pools could affect the results. Furthermore, although this study included information on up to 10,898 participants, it is possible that we overlooked some associations because of a lack of statistical power. However, the generally quite narrow CIs suggest that it is less likely that we overlooked any noteworthy associations. Thus, the public health relevance of vitamin D for adiposity changes is questionable. Though we had information on several possible confounders, unknown or residual confounding may still be operating. In this context, both measures of physical activity and dietary intake are known to be subject to considerable imprecision [52]. Furthermore, it could have been relevant to adjust for vitamin supplement use, but we did not have information available in all cohorts. The four SNP-scores were based on information from many of the established BMI, WC and WHR_BMI_ associated variants identified to date, however, these variants only explain a small amount of the total variation in obesity measures [53]. Thus, one could argue that it would be more appropriate to investigate interaction between phenotype at baseline (i.e. BW or WC) and 25(OH)D in relation to the subsequent change in the same measure. However, while this method is likely to capture a larger portion of the total genetic predisposition to obesity, it is also clear that a somewhat different question would be answered, because this method would not provide a separation of genetically and environmentally caused obesity. Likewise, the use of genetic predisposition scores based on genetic variants associated with current status of adiposity in cross-sectional GWAS may not be applicable when analyzing prospective change in BW and WC. In this regard, we have previously shown that although these genetic risk scores are strongly associated with a higher cross-sectional status of BW and WC, they are not generally associated with ΔBW or ΔWC [47]. This lack of association is consistent with the findings in the present study, though we did find an inverse association between WHR_BMI_ SNPs and ΔBW. Similarly, a recently published study found evidence for a contradictory inverse association between a high number of BMI-associated risk-alleles and less weight gain after middle-age, while a more expected increase in weight gain was observed in younger age. These and our results may indicate a possible disagreement between genetic susceptibility for obesity in different age groups across lifespan [54]. Yet, a general lack of a main effect for the genetic risk scores on changes in anthropometry does not preclude a possible interaction effect with for example 25(OH)D.

In addition, the estimates from the SNP-score × 25(OH)D interaction analyses can be described as the average interaction effect per additional adiposity associated risk allele. Thus, for the method to be suitable for capturing interaction effects of multiple SNPs, there needs to be a general consistency between the direction of cross-sectional adiposity-related main effect of the included SNPs and the direction of the SNP × 25(OH)D interaction effect in relation to subsequent change in adiposity. This assumption is not necessarily correct, and the limited findings with respect to the interaction analysis could in theory be explained by this.

The cohort samples available for analysis in this study may not be representative of the general population as some participants were excluded due to missing information on 25(OH)D, anthropometric outcomes, SNPs and/or covariates. However, as it is the effect of 25(OH)D on anthropometric changes which should be representative, and not necessarily the examined populations in general, we see no obvious reason why the results should not be the same in the respective background populations. Finally, our analyses were conducted in three cohorts of white-European descent and results may not generalize to other ethnic groups.

In conclusion, using data from three large prospective cohorts of North-Europeans, our study shows no association between 25(OH)D and subsequent change in BW or WC. Thus, due to the narrow CIs, our results suggest that any association between 25(OH)D and changes in measures of adiposity is absent or marginal. Furthermore, our results suggests that there is either no or very limited interaction with genetic predisposition to adiposity.

## Supporting Information

S1 Fig Annual change in waist circumference (mm/y) adjusted for concurrent weight change per 10 nmol/L higher 25-hydroxyvitamin D status. (DOCX)

S2 Fig Interaction between genetic predisposition scores and 25-hydroxyvitamin D in relation to subsequent change in waist circumference adjusted for concurrent weight change. (DOCX)

S1 Table Information on the 42 SNPs included in this study. (DOCX)

S2 Table SNP× 25-hydroxy vitamin D interaction in relation to annual change in body weight (g/y) per 10 nmol/L higher 25-hydroxy vitamin D. (DOCX)

S3 Table SNP× 25-hydroxy vitamin D interaction in relation to annual change in waist circumference (mm/y) per 10 nmol/L higher 25-hydroxy vitamin D. (DOCX)

S4 Table Annual change in body weight (ΔBW) and waist circumference (ΔWC) per 10 nmol/L higher 25-hydroxyvitamin D status in Inter99 (energy adjusted). (DOCX)

S5 Table Interaction between genetic predisposition scores and 25-hydroxyvitamin D in relation to subsequent annual change in body weight (ΔBW) and waist circumference (ΔWC) in inter99 (energy adjusted). (DOCX)
