# Supplementary material for: Serum 25-Hydroxyvitamin D Status and Longitudinal Changes in Weight and Waist Circumference: Influence of Genetic Predisposition to Adiposity
Source: PLoS One. 2016 Apr 14;11(4):e0153611. doi: 10.1371/journal.pone.0153611 (PMC4831693; doi:10.1371/journal.pone.0153611)
Supplement: S1 Fig — (DOCX) [file pone.0153611.s001.docx]

**S1 Figure. Annual change in waist circumference (mm/y) adjusted for concurrent weight change per 10 nmol/L higher 25-hydroxyvitamin D status.**

*Adjusted for baseline outcome, height, gender, age, smoking status, alcohol consumption, physical activity, education, menopausal status for women, season of blood draw and concurrent weight change .*

*Estimates were calculated in Inter99, 1958BC and NFBC1966 using linear regression and the results were subsequently meta-analyzed using a random effects approach.*
