# Supplementary material for: Serum 25-Hydroxyvitamin D Status and Longitudinal Changes in Weight and Waist Circumference: Influence of Genetic Predisposition to Adiposity
Source: PLoS One. 2016 Apr 14;11(4):e0153611. doi: 10.1371/journal.pone.0153611 (PMC4831693; doi:10.1371/journal.pone.0153611)
Supplement: S2 Fig — (DOCX) [file pone.0153611.s002.docx]

**S2 Figure. Interaction between genetic predisposition scores and 25-hydroxyvitamin D in relation to subsequent change in waist circumference adjusted for concurrent weight change.**

*Abbreviations: BMI score, sum of body mass index associated risk-alleles; WC score, sum of waist circumference associated risk-alleles; WHR score, sum of waist-hip ratio associated risk-alleles; Composite score, sum of SNP associated to all three phenotypes.*

*Results presented as annual change in waist circumference (mm/y) effect-modification for each additional risk-allele per 10 nmol/L higher 25-hydroxyvitamin D.*

*The study-specific SNP-score × 25-hydroxyvitamin D interactions were calculated using linear regression and corresponding meta-analysis results were derived using a random effects approach.*

*The results were adjusted for baseline measure of waist circumference, height, gender, age, smoking status, alcohol consumption, physical activity, education, menopausal status for women, season of blood draw and concurrent weight change.*
