# Supplementary material for: Serum 25-Hydroxyvitamin D Status and Longitudinal Changes in Weight and Waist Circumference: Influence of Genetic Predisposition to Adiposity
Source: PLoS One. 2016 Apr 14;11(4):e0153611. doi: 10.1371/journal.pone.0153611 (PMC4831693; doi:10.1371/journal.pone.0153611)
Supplement: S1 Table — (DOCX) [file pone.0153611.s003.docx]

| **S1 Table. Information on the 42 SNPs included in this study. The individual SNPs are sorted by refSNP (rs) number and grouped according to their associated trait** | | | | |
| --- | --- | --- | --- | --- |
| **Trait** | **SNP** | **Nearest gene** | **Risk allele** | **Other allele** |
|  |  |  |  |  |
| BMI | rs10838738 | *MTCH2* | G | A |
| BMI | rs10938397 ^1^ | *GNPDA2* | G | A |
| BMI | rs10968576 ^1^ | *LRRN6C* | G | A |
| BMI | rs11847697 ^1^ | *PRKD1* | T | C |
| BMI | rs12444979 ^1^ | *GPRC5B* | C | T |
| BMI | rs13107325 | *SLC39A8* | T | C |
| BMI | rs1424233 ^2^ | *MAF* | A | G |
| BMI | rs1514175 ^1^ | *TNNI3K* | T | C |
| BMI | rs1555543 ^1^ | *PTBP2* | C | A |
| BMI | rs17782313 ^1^ | *MC4R* | C | T |
| BMI | rs1805081 | *NPC1* | A | G |
| BMI | rs206936 ^1^ | *NUDT3* | G | A |
| BMI | rs2112347 ^1^ | *FLJ35779* | T | G |
| BMI | rs2241423 | *MAP2K5* | G | A |
| BMI | rs2287019 | *QPCTL* | C | T |
| BMI | rs2568958 | *NEGR1* | A | G |
| BMI | rs29941 | *KCTD15* | G | A |
| BMI | rs3810291 ^1^ | *TMEM160* | A | G |
| BMI | rs4929949 ^1^ | *RPL27A* | C | T |
| BMI | rs543874 ^1^ | *SEC16B* | G | A |
| BMI | rs713586 ^1, 2^ | *RBJ* | C | T |
| BMI | rs7647305 | *SFRS10* | C | T |
| BMI | rs9939609^1^ | *FTO* | A | T |
| BMI/WC | rs10146997 ^2^ | *NRXN3* | G | A |
| BMI/WC | rs1121980 ^1, 2^ | *FTO* | A | G |
| BMI/WC | rs7138803 | *FAIM2* | A | G |
| WC | rs12970134 | *MC4R* | A | G |
| WC | rs987237 | *TFAPB2* | G | A |
| WHR_BMI_ | rs1011731 | *DNM3-PIGC* | C | T |
| WHR_BMI_ | rs10195252 | *GRB14* | T | C |
| WHR_BMI_ | rs1055144 | *NFE2L3* | A | G |
| WHR_BMI_ | rs1294421 ^1^ | *LY86* | G | T |
| WHR_BMI_ | rs1443512 | *HOXC13* | A | C |
| WHR_BMI_ | rs2605100 | *LYPLAL1* | G | A |
| WHR_BMI_ | rs4823006 ^1^ | *ZNRF3-KREMEN1* | A | G |
| WHR_BMI_ | rs6784615 ^1^ | *NISCH-STAB1* | T | C |
| WHR_BMI_ | rs6795735 ^2^ | *ADAMTS9* | C | T |
| WHR_BMI_ | rs6861681 ^1^ | *CPEB4* | A | G |
| WHR_BMI_ | rs6905288 ^1^ | *VEGFA* | A | G |
| WHR_BMI_ | rs718314 ^2^ | *ITPR2-SSPN* | C | T |
| WHR_BMI_ | rs9491696 ^1^ | *RSPO3* | G | C |
| WHR_BMI_ | rs984222 ^1^ | *TBX15-WARS2* | G | C |
| *Abbreviations: BMI, body mass index; WC, waist circumference; WHR_BMI_, Waist-hip ratio adjusted for BMI. ^1^ Imputed SNP was used in NFBC1966*  *^2^ Proxy SNP was used for 1958BC* | | | | |
