# Supplementary material for: Serum 25-Hydroxyvitamin D Status and Longitudinal Changes in Weight and Waist Circumference: Influence of Genetic Predisposition to Adiposity
Source: PLoS One. 2016 Apr 14;11(4):e0153611. doi: 10.1371/journal.pone.0153611 (PMC4831693; doi:10.1371/journal.pone.0153611)
Supplement: S2 Table — (DOCX) [file pone.0153611.s004.docx]

| **S2 Table. SNP× 25-hydroxy vitamin D interaction in relation to annual change in body weight (g/y) per 10 nmol/L higher 25-hydroxy vitamin D. The results are sorted by refSNP (rs) number and grouped according to their associated trait** | | | | | | | | | |
| --- | --- | --- | --- | --- | --- | --- | --- | --- | --- |
|  |  | ***Inter99*** | | ***1958BC*** | | ***NFBC1966*** | | **Overall** | |
| **Trait** | **SNP** | **β^1^** | **P** | **Β** | **P** | **β** | **P** | **β** | **P** |
| BMI | rs10838738 | 2.9 | 0.75 | 11.1 | 0.40 | 1.1 | 0.92 | 3.9 | 0.51 |
| BMI | rs10938397 | 21.6 | 0.01 | -6.0 | 0.65 | -9.4 | 0.36 | 3.1 | 0.77 |
| BMI | rs10968576 | 3.5 | 0.71 | 46.0 | 0.00 | 0.2 | 0.99 | 14.8 | 0.25 |
| BMI | rs11847697 | -5.6 | 0.78 | -26.9 | 0.38 | 27.5 | 0.56 | -7.6 | 0.64 |
| BMI | rs12444979 | -24.4 | 0.06 | 16.5 | 0.37 | 0.5 | 0.97 | -5.1 | 0.67 |
| BMI | rs13107325 | -24.8 | 0.28 | -38.1 | 0.12 | 12.9 | 0.78 | -26.1 | 0.09 |
| BMI | rs1424233 | -1.3 | 0.88 | 25.8 | 0.05 | 9.1 | 0.34 | 8.6 | 0.23 |
| BMI | rs1514175 | 5.3 | 0.53 | -3.7 | 0.78 | -4.1 | 0.68 | 0.4 | 0.95 |
| BMI | rs1555543 | 8.1 | 0.35 | 1.8 | 0.89 | -7.2 | 0.45 | 1.4 | 0.81 |
| BMI | rs17782313 | 10.5 | 0.29 | 19.5 | 0.19 | -11.9 | 0.34 | 5.6 | 0.52 |
| BMI | rs1805081 | -3.7 | 0.66 | 26.1 | 0.04 | -6.2 | 0.52 | 3.4 | 0.71 |
| BMI | rs206936 | 2.3 | 0.84 | 4.5 | 0.77 | -11.8 | 0.31 | -2.6 | 0.72 |
| BMI | rs2112347 | 16.3 | 0.05 | 9.7 | 0.46 | 4.6 | 0.64 | 11.1 | 0.05 |
| BMI | rs2241423 | 1.3 | 0.90 | 3.4 | 0.82 | -2.3 | 0.86 | 0.6 | 0.93 |
| BMI | rs2287019 | -19.6 | 0.07 | 20.1 | 0.23 | 7.3 | 0.53 | 0.5 | 0.97 |
| BMI | rs2568958 | -12.2 | 0.17 | -13.2 | 0.32 | 6.9 | 0.49 | -5.6 | 0.40 |
| BMI | rs29941 | -5.5 | 0.55 | -9.1 | 0.50 | -4.9 | 0.61 | -6.0 | 0.32 |
| BMI | rs3810291 | -0.3 | 0.97 | -41.1 | 0.00 | 10.6 | 0.32 | -8.8 | 0.52 |
| BMI | rs4929949 | -2.9 | 0.73 | 10.3 | 0.41 | -1.3 | 0.90 | 0.4 | 0.95 |
| BMI | rs543874 | 25.5 | 0.01 | -22.6 | 0.15 | -3.2 | 0.79 | 1.6 | 0.91 |
| BMI | rs713586 | 5.4 | 0.52 | -10.0 | 0.43 | -1.1 | 0.90 | 0.1 | 0.99 |
| BMI | rs7647305 | -9.6 | 0.40 | -3.0 | 0.85 | 3.3 | 0.80 | -3.8 | 0.61 |
| BMI | rs9939609 | -11.3 | 0.20 | -4.5 | 0.74 | -4.8 | 0.62 | -7.6 | 0.19 |
| BMI/WC | rs10146997 | 12.4 | 0.22 | 2.8 | 0.86 | 1.8 | 0.88 | 6.8 | 0.32 |
| BMI/WC | rs1121980 | -9.5 | 0.29 | -0.8 | 0.95 | -7.7 | 0.44 | -7.0 | 0.24 |
| BMI/WC | rs7138803 | 0.8 | 0.93 | -8.8 | 0.50 | 3.7 | 0.70 | -0.1 | 0.99 |
| WC | rs12970134 | 11.4 | 0.23 | 15.6 | 0.27 | -4.8 | 0.69 | 7.5 | 0.26 |
| WC | rs987237 | 1.9 | 0.87 | -12.7 | 0.46 | -9.0 | 0.44 | -5.1 | 0.49 |
| WHR_BMI_ | rs1011731 | -11.6 | 0.18 | -1.5 | 0.91 | 3.4 | 0.72 | -4.2 | 0.46 |
| WHR_BMI_ | rs10195252 | 2.5 | 0.76 | -11.7 | 0.37 | 18.0 | 0.07 | 4.3 | 0.58 |
| WHR_BMI_ | rs1055144 | -11.6 | 0.30 | -13.9 | 0.38 | -15.6 | 0.13 | -13.8 | 0.05 |
| WHR_BMI_ | rs1294421 | -19.9 | 0.02 | 25.0 | 0.05 | 6.8 | 0.49 | 2.8 | 0.83 |
| WHR_BMI_ | rs1443512 | 3.2 | 0.75 | 3.2 | 0.84 | 10.7 | 0.32 | 6.1 | 0.36 |
| WHR_BMI_ | rs2605100 | -6.4 | 0.49 | -7.2 | 0.60 | 10.1 | 0.32 | -0.7 | 0.91 |
| WHR_BMI_ | rs4823006 | -7.8 | 0.36 | -10.0 | 0.45 | 1.5 | 0.88 | -5.2 | 0.38 |
| WHR_BMI_ | rs6784615 | 0.0 | 1.00 | -5.5 | 0.83 | -8.1 | 0.78 | -3.4 | 0.80 |
| WHR_BMI_ | rs6795735 | -4.9 | 0.56 | 1.3 | 0.92 | -7.4 | 0.44 | -4.6 | 0.42 |
| WHR_BMI_ | rs6861681 | -0.9 | 0.92 | 34.0 | 0.02 | -0.4 | 0.97 | 8.6 | 0.39 |
| WHR_BMI_ | rs6905288 | -11.1 | 0.18 | 21.6 | 0.10 | 12.3 | 0.36 | 5.6 | 0.60 |
| WHR_BMI_ | rs718314 | 6.5 | 0.49 | 16.0 | 0.28 | -4.8 | 0.65 | 4.1 | 0.52 |
| WHR_BMI_ | rs9491696 | 10.0 | 0.23 | 7.1 | 0.58 | 1.6 | 0.87 | 6.6 | 0.25 |
| WHR_BMI_ | rs984222 | -3.3 | 0.71 | -5.9 | 0.66 | 7.7 | 0.47 | -0.3 | 0.96 |
| *Abbreviations: BMI, body mass index; WC, waist circumference; WHR_BMI_, waist-hip ratio adjusted for BMI.*  *^1^ The study-specific SNP-score × 25-hydroxyvitamin D interactions were calculated using linear regression and meta-analysis results were derived using a random effects approach. The results were adjusted for baseline body weight, height, gender, age, smoking status, alcohol consumption, physical activity, education, menopausal status for women and season of blood draw.* | | | | | | | | | |
