# Supplementary material for: Serum 25-Hydroxyvitamin D Status and Longitudinal Changes in Weight and Waist Circumference: Influence of Genetic Predisposition to Adiposity
Source: PLoS One. 2016 Apr 14;11(4):e0153611. doi: 10.1371/journal.pone.0153611 (PMC4831693; doi:10.1371/journal.pone.0153611)
Supplement: S3 Table — (DOCX) [file pone.0153611.s005.docx]

| **S3 Table. SNP× 25-hydroxy vitamin D interaction in relation to annual change in waist circumference (mm/y) per 10 nmol/L higher 25-hydroxy vitamin D. The results are sorted by refSNP (rs) number and grouped according to their associated trait** | | | | | | | |
| --- | --- | --- | --- | --- | --- | --- | --- |
|  |  | ***Inter99*** | | ***NFBC1966*** | | **Overall** | |
| **Trait** | **SNP** | **β ^1^** | **P** | **Β** | **P** | **Β** | **P** |
| BMI | rs10838738 | 0.09 | 0.45 | -0.08 | 0.43 | 0.00 | 0.97 |
| BMI | rs10938397 | 0.12 | 0.25 | -0.09 | 0.39 | 0.02 | 0.89 |
| BMI | rs10968576 | -0.08 | 0.50 | -0.04 | 0.66 | -0.06 | 0.44 |
| BMI | rs11847697 | -0.26 | 0.29 | 0.25 | 0.61 | -0.16 | 0.48 |
| BMI | rs12444979 | -0.34 | 0.03 | -0.11 | 0.51 | -0.22 | 0.05 |
| BMI | rs13107325 | -0.10 | 0.72 | 0.36 | 0.46 | 0.02 | 0.95 |
| BMI | rs1424233 | -0.10 | 0.34 | 0.17 | 0.10 | 0.04 | 0.79 |
| BMI | rs1514175 | 0.03 | 0.79 | -0.15 | 0.14 | -0.06 | 0.48 |
| BMI | rs1555543 | 0.03 | 0.80 | 0.10 | 0.34 | 0.06 | 0.39 |
| BMI | rs17782313 | 0.27 | 0.03 | -0.17 | 0.18 | 0.05 | 0.82 |
| BMI | rs1805081 | -0.05 | 0.65 | -0.03 | 0.79 | -0.04 | 0.61 |
| BMI | rs206936 | 0.11 | 0.45 | -0.18 | 0.13 | -0.05 | 0.74 |
| BMI | rs2112347 | 0.08 | 0.42 | 0.04 | 0.71 | 0.06 | 0.41 |
| BMI | rs2241423 | 0.03 | 0.83 | 0.01 | 0.93 | 0.02 | 0.83 |
| BMI | rs2287019 | -0.13 | 0.33 | -0.05 | 0.67 | -0.09 | 0.33 |
| BMI | rs2568958 | 0.03 | 0.81 | 0.01 | 0.94 | 0.02 | 0.83 |
| BMI | rs29941 | -0.04 | 0.71 | 0.02 | 0.88 | -0.01 | 0.90 |
| BMI | rs3810291 | -0.05 | 0.66 | 0.07 | 0.56 | 0.01 | 0.91 |
| BMI | rs4929949 | -0.05 | 0.64 | 0.01 | 0.95 | -0.02 | 0.78 |
| BMI | rs543874 | 0.35 | 0.01 | -0.10 | 0.42 | 0.12 | 0.59 |
| BMI | rs713586 | 0.07 | 0.51 | -0.06 | 0.53 | 0.00 | 0.99 |
| BMI | rs7647305 | 0.02 | 0.88 | 0.18 | 0.19 | 0.10 | 0.29 |
| BMI | rs9939609 | -0.08 | 0.48 | -0.10 | 0.34 | -0.09 | 0.24 |
| BMI/WC | rs10146997 | 0.27 | 0.04 | -0.03 | 0.81 | 0.12 | 0.43 |
| BMI/WC | rs1121980 | -0.14 | 0.21 | -0.13 | 0.21 | -0.14 | 0.08 |
| BMI/WC | rs7138803 | 0.06 | 0.57 | 0.04 | 0.70 | 0.05 | 0.51 |
| WC | rs12970134 | 0.25 | 0.03 | -0.07 | 0.57 | 0.09 | 0.57 |
| WC | rs987237 | 0.12 | 0.39 | -0.08 | 0.49 | 0.01 | 0.94 |
| WHR_BMI_ | rs1011731 | 0.00 | 0.97 | 0.01 | 0.93 | 0.00 | 0.97 |
| WHR_BMI_ | rs10195252 | 0.03 | 0.75 | 0.19 | 0.07 | 0.11 | 0.16 |
| WHR_BMI_ | rs1055144 | -0.32 | 0.02 | -0.11 | 0.29 | -0.20 | 0.05 |
| WHR_BMI_ | rs1294421 | -0.26 | 0.02 | 0.05 | 0.63 | -0.10 | 0.50 |
| WHR_BMI_ | rs1443512 | -0.15 | 0.23 | 0.07 | 0.53 | -0.03 | 0.76 |
| WHR_BMI_ | rs2605100 | -0.01 | 0.91 | 0.19 | 0.08 | 0.09 | 0.36 |
| WHR_BMI_ | rs4823006 | -0.05 | 0.61 | 0.01 | 0.90 | -0.02 | 0.78 |
| WHR_BMI_ | rs6784615 | -0.08 | 0.73 | -0.06 | 0.84 | -0.07 | 0.69 |
| WHR_BMI_ | rs6795735 | 0.04 | 0.72 | -0.07 | 0.49 | -0.02 | 0.80 |
| WHR_BMI_ | rs6861681 | -0.05 | 0.66 | -0.01 | 0.91 | -0.03 | 0.70 |
| WHR_BMI_ | rs6905288 | -0.17 | 0.10 | 0.08 | 0.57 | -0.06 | 0.61 |
| WHR_BMI_ | rs718314 | 0.07 | 0.54 | -0.06 | 0.61 | 0.00 | 0.97 |
| WHR_BMI_ | rs9491696 | 0.20 | 0.05 | -0.09 | 0.38 | 0.06 | 0.70 |
| WHR_BMI_ | rs984222 | -0.05 | 0.66 | 0.03 | 0.80 | -0.01 | 0.89 |
| *Abbreviations: BMI, body mass index; WC, waist circumference; WHR_BMI_, waist-hip ratio adjusted for BMI. ^1^ The study-specific SNP-score × 25-hydroxyvitamin D interactions were calculated using linear regression and meta-analysis results were derived using a random effects approach. The results were adjusted for baseline waist circumference, height, gender, age, smoking status, alcohol consumption, physical activity, education, menopausal status for women and season of blood draw.* | | | | | | | |
