# Supplementary material for: Serum 25-Hydroxyvitamin D Status and Longitudinal Changes in Weight and Waist Circumference: Influence of Genetic Predisposition to Adiposity
Source: PLoS One. 2016 Apr 14;11(4):e0153611. doi: 10.1371/journal.pone.0153611 (PMC4831693; doi:10.1371/journal.pone.0153611)
Supplement: S4 Table — (DOCX) [file pone.0153611.s006.docx]

| **S4 Table: Annual change in body weight (ΔBW) and waist circumference (ΔWC) per 10 nmol/L higher 25-hydroxyvitamin D status in Inter99 (energy adjusted).** | | | | | |
| --- | --- | --- | --- | --- | --- |
|  | n | β | 95% CI | | P |
| ΔBW | 3,798 | 1.366 | -10.812 | 13.544 | 0.826 |
| ΔWC | 3,295 | -0.008 | -0.159 | 0.143 | 0.917 |
| *Results presented as annual weight change (g/y) or waist change (mm/y) effect-modification for each additional risk-allele per 10 nmol/L higher 25-hydroxyvitamin D.*  *Adjusted for baseline outcome, height, gender, age, smoking status, alcohol consumption, physical activity, education, menopausal status for women, season of blood draw and total energy intake.* | | | | | |
