# Supplementary material for: Serum 25-Hydroxyvitamin D Status and Longitudinal Changes in Weight and Waist Circumference: Influence of Genetic Predisposition to Adiposity
Source: PLoS One. 2016 Apr 14;11(4):e0153611. doi: 10.1371/journal.pone.0153611 (PMC4831693; doi:10.1371/journal.pone.0153611)
Supplement: S5 Table — (DOCX) [file pone.0153611.s007.docx]

| **S5 Table. Interaction between genetic predisposition scores and 25-hydroxyvitamin D in relation to subsequent annual change in body weight (ΔBW) and waist circumference (ΔWC) in inter99 (energy adjusted)** | | | | | |
| --- | --- | --- | --- | --- | --- |
| **ΔBW** | N | β | 95% CI | | P |
| BMI-score | 2605 | -1.232 | -5.431, | 2.966 | 0.565 |
| WC-score | 3272 | 2.233 | -6.765, | 11.231 | 0.627 |
| WHR-score | 3058 | -4.651 | -9.651, | 0.349 | 0.068 |
| Composite-score | 2162 | -2.478 | -6.230, | 1.274 | 0.195 |
| **ΔWC** |  |  |  |  |  |
| BMI-score | 2243 | -0.001 | -0.054, | 0.051 | 0.956 |
| WC-score | 2830 | 0.122 | 0.009, | 0.235 | 0.034 |
| WHR-score | 2652 | -0.056 | -0.119, | 0.007 | 0.081 |
| Composite-score | 1863 | -0.027 | -0.075, | 0.020 | 0.254 |
| *Abbreviations: BMI-score, sum of body mass index associated risk-alleles; WC-score, sum of waist circumference associated risk-alleles; WHR-score, sum of waist-hip ratio associated risk-alleles; Composite-score, sum of SNP associated to all three phenotypes. Results presented as annual weight change (g/y) or waist change (mm/y) effect-modification for each additional risk-allele per 10 nmol/L higher 25-hydroxyvitamin D.  The study-specific SNP-score × 25-hydroxyvitamin D interactions were calculated using linear regression The results were adjusted for baseline measure of body weight, height, gender, age, smoking status, alcohol consumption, physical activity, education, menopausal status for women, season of blood draw and total energy intake.* | | | | | |
